# Marmots and *Yersinia pestis* Strains in Two Plague Endemic Areas of Tien Shan Mountains

**DOI:** 10.3389/fvets.2019.00207

**Published:** 2019-07-04

**Authors:** Gulmira Sariyeva, Gulnara Bazarkanova, Ravshambek Maimulov, Sabirzhan Abdikarimov, Berzhan Kurmanov, Aigul Abdirassilova, Anton Shabunin, Zaurbek Sagiyev, Aigul Dzhaparova, Ziyat Abdel, Raikhan Mussagaliyeva, Serge Morand, Vladimir Motin, Michael Kosoy

**Affiliations:** ^1^Department of Natural Sciences, Issyk-Kul State University, Karakol, Kyrgyzstan; ^2^Karakol Anti-plague Department, Republic Center of Quarantine and Dangerous Infections, Karakol, Kyrgyzstan; ^3^Republic Center of Quarantine and Dangerous Infections, Bishkek, Kyrgyzstan; ^4^Reference-Laboratory, Kazakh Scientific Center of Quarantine and Zoonotic Diseases, Almaty, Kazakhstan; ^5^Institute of Evolutionary Sciences, University of Montpellier, Montpellier, France; ^6^Department of Microbiology and Immunology, Medical Branch, University of Texas, Galveston, TX, United States; ^7^Division of Vector-Borne Diseases, Centers for Disease Control and Prevention, Fort Collins, CO, United States

**Keywords:** grey marmot, ectoparasites, plague, rodent, Kyrgyzstan, *Yersinia pestis*

## Abstract

The main purpose of this study was to clarify the role of gray marmots (*Marmota baibacina*) in the long-term maintenance of highly virulent strains of *Yersinia pestis* in two plague endemic foci of the Tien Shan Mountains in Kyrgyzstan. We present data from regular observations of populations of *M. baibacina* and small rodents cohabiting with marmots in the mountainous grasslands of the Sari-Dzhas (east of Issyk-Kul Lake) and the Upper-Naryn (south of Issyk-Kul Lake) natural foci. During 2012–2017, an abundance of marmots and their ectoparasites (fleas and ticks) was significantly higher in Upper-Naryn comparing to Sari-Dzhas, although there were no differences in a number and diversity of small rodents cohabiting with marmots. The plague bacterium was detected either in marmots or in their ectoparasites collected during 4 of 6 years of observation in Sari-Dzhas and during 2 of 4 years of observation in Upper-Naryn. Plague was found in three sectors situated closely to each other in Sari-Dzhas and in 1 of 8 repeatedly surveyed sectors in Upper-Naryn. During 6 years, we isolated 9 strains of *Y. pestis* from marmots, two from their fleas *Oropsylla silantiewi*, one from an unidentified tick, and one from the gray hamster (*Cricetulus migratorius*). All plague strains isolated from the rodents and their ectoparasites in this study were similar to *Antiqua* biovar specific for marmots. The results indicate that plague can circulate continuously in the Tien Shan Mountains in populations of gray marmots and their ectoparasites with a facultative involvement of other rodent species after significant changes in rodent communities that happened in Kyrgyzstan during the previous two decades. The simultaneous field survey of two natural foci of plague, Sari-Dzhas, and Upper-Naryn, would be important for further analysis of circulation of *Y. pestis* strains belonging to *Antiqua* biovar in the Tien Shan Mountains.

## Introduction

One of the most active plague endemic areas in the world is located in Kyrgyzstan, Central Asia. There are two independent foci of plague in the Issyk-Kul province: the Sari-Dzhas (east of Issyk-Kul Lake) and the Upper-Naryn (south of Issyk-Kul Lake) sub-regions of the Tien Shan natural focus of plague (1, 2). Several plague outbreaks involving natural vectors, including the gray marmots (*Marmota baibacina*) and other rodents, have been registered there since 1907 (3). Most of the 5,000 km^2^ Sari-Dzhas natural plague focus is located in Kyrgyzstan (4,250 km^2^), with a part extending to Kazakhstan (750 km^2^). The 8,000 km^2^ Upper-Naryn area is located in the Naryn and Issyk-Kul regions of Kyrgyzstan.

The first plague outbreak with high human mortality was recorded in the Kyrgyzstan part of Tien Shan focus in 1907, and the last big outbreak of plague occurred in 1928 in the Bash-Kaindi settlement of Atbashi district resulting in 54 deaths (Upper-Naryn mezofocus) (4). Sporadic human cases were also seen in 1965 and 1982. In 2013, a 15-year-old boy who ate marmot meat died from plague in Kyrgyzstan. The Kyrgyz Ministry of Health established a temporal quarantine in parts of the country's mountainous northeast (5), where the risk of plague is well-known for a century. The endemic areas were investigated previously starting from 1942, and the last activity of plague was reported there in 1983 (4, 6, 7).

Gray marmots (*M. baibacina*) are well-known hosts of multiple zoonotic diseases, out of which plague presents the greatest danger to people (8–10). The gray marmots prefer grasslands and shrubs and avoid woodlands, including even the forest edges and forest-steppe areas with a tree cover <10% (11). Marmot fleas are actively involved in transmission of plague pathogen between animals (12–14). Marmots can host the following species of fleas: *Oropsylla silantiewi, Rhadinopsylla li ventricosa, Ceratophyllus lebedewi*, and *Pulex irritans*; the first two flea species are host-specific for marmots (15, 16).

Human activities, such as agriculture, hunting, recreation, and degradation of natural habitats, may dramatically influence plague manifestations (17). Changes in landscape potentially can lead to altering the rodent and flea communities that in turn affect plague transmission cycle (18). Plague in Asian natural systems is commonly spatially stable and corresponds with distribution of primary rodent hosts that can support a strong association of specific strains of *Yersinia pestis* in mammalian communities (19). Marmots in mountainous plague foci of Central Asia carry a specific strain of *Y. pestis* (20). Disturbance of evolutionary relations between plague pathogen and their rodent hosts, such as marmots, may result in unpredictable dynamics of the infection.

The collapse of the Soviet Union and subsequent separation of anti-plague station in Kyrgyzstan from the federal anti-plague system led to dramatic reduction of plague investigations and plague control measures. During the last couple of decades, the density of marmots was significantly reduced as a result of chemical suppression in the 1960s, increased hunting of marmots, habitat destruction, and climate changes (6–8, 21). This raises the question whether the changes in rodent communities in these areas affected marmots playing a leading role in circulation of plague pathogen. Thus, the present study aimed to clarify the role of gray marmots in the long-term maintenance of the highly virulent strains of *Y. pestis* in two plague foci of the Tien Shan Mountains in Kyrgyzstan. The human case in 2013 attracted attention to the situation that plague is endemic to the territory of Kyrgyzstan and the urgent need to obtain new information about the status of the epidemiological situation in these plague foci.

## Materials and Methods

### Fieldwork

In June–August 2012–2017, the Karakol Anti-Plague Department (KAPD) of the Republic Center for Quarantine and Dangerous Infections (RCQDI) organized field surveys within the Sari-Dzhas and Upper-Naryn foci. The fieldwork was organized as described by Weaver et al. (22) and Sariyeva et al. (6, 7). All work with wild and laboratory animals and plague strains was conducted in accordance with the regulations and protocols approved by the Ministry of Health of Kyrgyzstan (23) in 2015. The procedures were similar to those described by Aytkuluyev (24) and Ezhlova et al. (25). The animal work in the field was performed according to the Regulations approved by the State Agency for Environmental and Forest Protection of the Kyrgyz Republic (details of permits are presented in Table 1). Each permit was issued for collecting a certain number of animals (400 marmots, 300–400 rodents of other species, and excavating 10 nests of marmots and rodents to look for nest parasites) and used during a fixed period of time (from June 1 to August 30) in specific areas within plague foci.

**Table 1:** Numbers and dates of permits for trapping of animals for epizootological research in 2012–2017.

**Year**	**Sari-Dzhas**			**Upper Naryn**		
	**Number of license**	**Data of issue**	**Permitted number of animals**	**Number of license**	**Data of issue**	**Permitted number of animals**
2012	000078-KC	26.04.2012	400 marmots, 400 small rodents	–	–	–
2013	000110-KC	06.05.2013	400 marmots, 300 s.r.	000111-KC	06.05.2013	300 marmots, 300 small rodents
2014	000144-KC	06.05.2014	400 marmots, 300 s.r.	000145-KC	06.05.2014	400 marmots, 300 s.r.
2015	000168-KC	22.05.2015	400 marmots, 300 s.r.	000169-KC	22.05.2015	400 marmots, 300 s.r.
2016	000173-KC	17.05.2016	400 marmots, 300 s.r.	000174-KC	17.05.2016	400 marmots, 300 s.r.
2017	000003-KC	05.03.2017	400 marmots, 300 s.r.	000004-KC	05.03.2017	400 marmots, 300 s.r.

### Trapping Rodents

At the beginning of the annual anti-plague field work, zoologists visually estimated a number of marmots within study sites and accordingly planned trapping efforts in each sector (10 km^2^). The number of installed traps depends on the average density of marmot population in each sector. Around 8–10 traps were set next to marmot burrows each day at early morning. As the weather in May–June in high-altitude areas of Tien Shan is highly variable with snow, rains, and cold weather, the trapping was conducted during each sunny day. The marmot traps were set at the entrance of burrows and removed by evening. One trap remained at each burrow for half a day. The burrows for trapping were chosen based on external signs such as presence of fresh litter at the entrance, presence of freshly excavated and well-rammed entrances, grass coverage, presence of marmot footprints, and paths around the burrow. The traps used for capturing marmots were 20 cm wide and 8 cm high (Figure 5). The traps were attached to 50-cm-long metal rods with a strong metal chain. The rods are driven into the rocky soil to their full depth to prevent marmots or their predators from dragging the traps away. The metal parts were masked by horse manure to reduce smell of metal.

A number of small rodents, such as mice, voles, and hamsters, were estimated by counting animals captured by handmade snap traps set in transects (1, 25). The snap traps were also sprinkled with soil and baited with dry bread soaked in vegetable oil, vegetables, or fruit. We placed traps in the evening and checked them in the early morning. If there was a captured rodent, we transferred the trap to another spot. The predators were estimated by trapping and visual observations. Each trapped animal was morphologically identified by species. The captured marmots were euthanized by cervical dislocation (25). Then, the animal was wrapped in two linen sacs with label (date, place, number of sector, and name of catcher), the inlet was tightly wrapped so that the ectoparasites could not escape, and then the animal was placed in a plastic bag, in a canvas bag, and finally brought to camp.

### Parasitological Analysis

Ectoparasites of marmots were collected by combing the captured animals (“body” ectoparasites) and by collecting “off-host” ectoparasites from rodent burrows and nests (25). Ectoparasites were then identified using entomological keys (26) and placed in labeled glass tubes with ether. Inside burrows, dry grass bedding from the nesting chamber was carefully removed with a wire and was searched for fleas and other ectoparasites. Fleas of one species from a single animal were pooled and triturated in saline solution.

### Bacteriological Analysis

The triturated flea suspension was inoculated on Hottinger agar with pH 7.2 (27) (bacteriological approach). The inoculation pool consisted of 20 ectoparasites of one species, collected from the same sector during several days and stored before inoculation in glass tubes without ether. If the animal exhibited any pathological manifestation, the ectoparasites from the pool were inoculated individually. Identification of the isolated bacteria was carried out by standard microbiological microscopy of smears and by Pokrovsky's test for *Y. pestis* using both pseudo-tuberculosis bacterial phage and bacteriophage for *Y. pestis* (L-413-C) produced by the Kazakh Scientific Center for Quarantine and Zoonotic Diseases (KSCQZD). Additional diagnostic methods included serological indirect hemagglutination assay with both erythrocyte–immunoglobulin and erythrocyte–antigen diagnostic reagents produced by KSCQZD. The tissues of internal organs of sampled marmots (liver, lung, spleen, lymph nodes, and blood from the heart) were sterilely taken with forceps and immediately placed on an agar plate by directly touch. A Petri dish was divided into three segments to separately plate lungs, livers, and spleens. Each plate was marked and placed in an incubator at 37°C. We used individual and pool methods of inoculation. The individual method was used for ectoparasites collected from carcass of marmot or marmot with visual pathological abnormalities relevant to plague (pathology of inner organs, sick marmots). For inoculation of pooled tissue suspensions, we combined tissue pieces from five marmots collected on the same day from the same sector. Then, the organ suspension was inoculated on agar plate with a bacterial inoculating loop. After inoculation, tissue samples were placed in liquid nitrogen (Dewar flask) and transported to the laboratory in Karakol for further confirmation.

### Genetic Analysis

The genetic analysis was performed using two types of material: whole-genome DNA samples and MLVA fragments (Table 2). The 14 studied *Y. pestis* strains were obtained from the RCQDI collection. The strains were isolated during the last several years in the territory of Sari-Dzhas and Upper-Naryn plague foci (Table 2). The suspension of *Y. pestis* cultures was heated at 100°C for 20 min and centrifuged at 12,000 rpm for 2 min. This allowed the inactivation of the pathogens and the release of the DNA in the cells. The DNA samples were further used for genotyping. The control MLVA amplification fragments of the reference strains CO92, Pestoides F, KIM10+, and Nepal 516 of *Y. pestis* were obtained from the University of Texas Medical Branch, Texas, USA. In addition, nucleotide sequences of nine *Y. pestis* species (GenBank accession numbers: CP010023, CP010247, CP006751, CP009935, AE017042, CP006806, CP000308, CP006794, and CP002956) and three *Yersinia pseudotuberculosis* strains (GenBank accession numbers: CP009712, CP008943, and CP001048) were used for phylogenetic analysis. The studied strains belonging to the *Y. pestis* species was confirmed using the “Pest-Quest” PCR assay (“Master-Gene” Limited Liability Partnership, Almaty, Kazakhstan). The genotyping of several *Y. pestis* strains was performed by the methods of Multi-Locus VNTR Analysis (MLVA) and Melt Analysis of Mismatch Amplification Mutation Assay (Melt-MAMA). For MLVA assay, seven VNTR (Variable Number Tandem Repeats) loci were studied using conventional PCR and agarose gel electrophoresis as described by Le Flèche et al. (28). For Melt-MAMA assay, three sets of PCR primers designed and produced by KSCQZD were used to identify three SNP loci, described by Morelli et al. (29). The selected SNP loci allowed differentiation of four branches of *Y. pestis* bv. *Antiqua* (0.ANT1, 0.ANT2, 0.ANT3, and 3.ANT). The Melt-MAMA analysis was performed as described by Birdsell et al. (30). Each primer set consisted of two forward primers (“ancestral” and “derived”) and a universal reverse primer. One of the forward primers was linked to the so-called GC-clamp, three to four repeats of a GGGGC motif, which increased the melting temperature of the corresponding amplicons and made it possible to differentiate the alleles. Phylogenetic analysis of the studied strains was carried out using the PAUP 4.0 software and the UPGMA (Unweighted Pair Group Method with Arithmetic Mean) algorithm.

**Table 2:** Strains of *Y. pestis* isolated in the Sari-Dzhas and Upper-Naryn plague foci used for DNA analysis.

**Strain code**	**Object, site, and period of strain isolation**
KG-1	Human outbreak, the Sari-Dzhas, 2013
KG-2	Human outbreak, the Sari-Dzhas, 2013
KG-3	Gray marmot, the Sari-Dzhas, 2014
KG-4	Gray marmot, the Upper-Naryn natural plague focus, Ishtyk-Akshiyrak site, 2015
KG-5	Gray marmot, the Upper-Naryn natural plague focus, Ishtyk-Akshiyrak site, 1963
KG-6	Ectoparasites, there was a suspicion that this strain was *Y. pseudotuberculosis*, 1962
KG-7	Gray marmot carcass, the Sari-Dzhas natural plague focus, Enylchek-Kaindy site, 2016
KG-8	Ectoparasites—ticks from the carcass of gray marmot, the Sari-Dzhas natural plague focus, Enylchek-Kaindy site, 2016
KG-9	Ectoparasites—fleas from the carcass of gray marmot, the Sari-Dzhas natural plague focus, Enylchek-Kaindy site, 2016
KG-10	Gray marmot, the Sari-Dzhas natural plague focus, Enylchek-Kaindy site, 2016
KG-11	Gray marmot, the Upper-Naryn natural plague focus, Ishtyk-Akshiyrak site, 1957
KG-12	Gray marmot, the Upper-Naryn natural plague area, Ishtyk-Akshiyrak site, 1962
KG-13	Gray marmot, the Upper-Naryn natural plague area, Ishtyk-Akshiyrak site, 1960
KG-14	Gray marmot, the Upper-Naryn natural plague area, Ishtyk-Akshiyrak site, 1983

### Mapping

The map of Sari-Dzhas and Upper-Naryn areas of plague was prepared using GIS MapInfo Professional 7.8. For their construction, we used the geographic objects digitized from topographic maps of a scale of 1:100,000, as well as the Digital Elevation Model GDEM2. The maps are constructed in UTM—the Mercater projection (WGS 84). The boundaries of the foci are plotted along the boundaries of the sectors (21).

## Results

### Sari-Dzhas Plague Focus

#### Rodents

**Figure 1:**
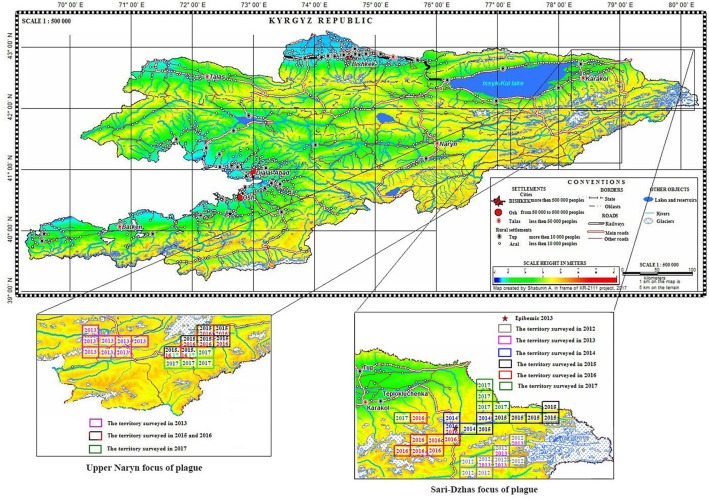
Localization of the Sari-Dzhas and Upper-Naryn natural plague foci and conducted field activities in 2012–2017 (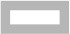
—the territory survived in 2012; 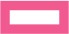
—the territory survived in 2013; 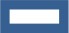
—the territory survived in 2014; 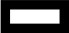
—the territory survived in 2015; 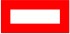
—the territory survived in 2016; 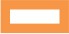
—the territory survived in 2017).

Overall, in the Sari-Dzhas plague focus for the period 2012 to 2017, we surveyed a total of 3,700 km^2^, out of which 500 km^2^ were surveyed repeatedly in 2012, 2013, 2014, and 2016 (Figure 1; Table 4). In total, we trapped and analyzed 972 marmots (*M. baibacina*), 442 narrow-headed voles (*Microtus gregalis*), 61 wood mouse (*Apodemus uralensis*), 40 gray dwarf hamsters (*Cricetulus migratorius*), and a small number of other mammals: the large-eared pika (*Ochotona macrotis*), the hare-tolai (*Lepus tolai*), and the beech marten (*Martes foina*) (Table 3). Additionally, we found carcasses of six dead rodents.

**Table 3:** Field survey of the Sari-Dzhas and Upper Naryn sites of the Tien Shan high-altitude focus of plague in 2012–2017.

**Focus**	**Year**	**Month**	**Area and number of sectors**	**Number of caught marmots**	**Number of other mammals trapped**	**Number of collected ectoparasites**	***Y. pestis* isolated from marmots**	***Y. pestis* isolated from ectoparasites**	***Y. pestis* isolated from other rodents**
Sari-Dzhas	2012	June–July	800 km^2^, 8 sectors	218	*Microtus gregalis*−42, *Apodemus uralensis*−47, *Cricetulus migratorius*−12 + carcass, *Lepus tolai*−2 (total 103)	240 fleas, 459 ticks	5	–	1 (gray hamster)
	2013	June	400, 4 sectors	70	*Microtus gregalis*−30, *Apodemus uralensis*−6, *Cricetulus migratorius*−10 (total 46)	10 fleas, 137 ticks	–	–	–
	2014	July	400, 4 sectors	197	*Microtus gregalis*−42, *Ochotona macrotis*−1, *Apodemus uralensis*−1, *Cricetulus migratorius*−11 (total 55)	84 fleas, 155 ticks, 6 lice	–	1 from fleas	–
	2015	July–August	600, 6 sectors	177	*Microtus gregalis*−129, *Martes foina*−5 (total 134)	175 fleas, 42 ticks, 16 lice	–	–	–
	2016	June–July	800, 8 sectors	180	*Microtus gregalis*−124, *Apodemus uralensis*−3, *Cricetulus migratorius*−1 (total 128)	145 fleas, 344 ticks, 9 lice	2	1 from ticks	–
	2017	June–July	700, 7 sectors	130	*Microtus gregalis*−75, *Apodemus uralensis*−4 (total 79)	124 fleas, 15 ticks, 125 lice	–	–	–
Upper Naryn	2013	June–August	800, 8 sectors	154	*Microtus gregalis*−47, *Cricetulus migratorius*−13, *Mustela eversmanni*−4 (total 64)	268 fleas, 1045 ticks, 127 lice	–	–	–
	2015	June–July	800, 8 sectors	260	*Microtus gregalis*−107, *Lepus tolai*−2, *Cuon alpinus*−1, *Mustela eversmanni*−2 (total 112)	489 fleas, 113 ticks, 52 lice	2	–	–
	2016	June–August	700, 7 sectors	190	*Microtus gregalis*−150, *Alticola argentatus*−1, *Vulpes vulpes*−1 (total 152)	261 fleas, 233 ticks, 2 lice	–	1 from fleas	–
	2017	June–July	600, 6 sectors	224	*Microtus gregalis*−38, *Lepus tolai*−2, *Vulpes vulpes*−2 (total 42)	311 fleas, 593 ticks, 38 lice	–	–	–

From all sampled marmots, we collected and tested 778 fleas, 1,152 ticks, and 156 lice (Table 3). The average number of sampled marmots ranged from 17 to 27 animals per sector in 2012 and 2013 (Table 4). The highest number of trapped marmots (49 ± 7.62) was recorded in 2014 in a forested area with preferable conditions such as vegetation and humidity. In 2015–2017, the number of collected marmots was similar to 2012. Among small rodents, the most common species was *M. gregalis*, which is widely distributed across all observed territories (Tables 3, 4). The density of population of *M. gregalis* and *A. uralensis* varied significantly. A reverse correlation between abundance of these two species was observed. A high number of *M. gregalis* was associated with limited or no *A. uralensis* (Table 4).

**Table 4:** The number of trapped mammals in open stations of the Sari-Dzhas natural plague focus in 2012–2017.

**Years of observation**	**#sector**	**Time period of observation**	***Marmota baibacina*** **(trapped)**	***Marmota baibacina*** **(carcass)**	***Microtusgregalis***	***Apodemus uralensis***	***Ochotona macrotis***	***Cricetulus migratorius***	***Lepus tolai***	***Martes foina***	**Isolated strain of *Y. pestis***
2012	3224406334	29 May−3 June	28		1	1			1	**–**	
	3224407512	29 May−2 June	18		1	3		1			
	3124407521	6–11 June	43		–	14		3			
	3124406343	6–11 June	26	1	–	6		4 + carcass			
	3124406344	13–15 June	18		–	6		3			
	3124406433	14–15 June	7		–	–					
	3124406342	18–29 June	53	2^*^	33	12		1^*^	1		**5**
	3124406413	19–26 June	25		7	5		–			
	**Average:**		**27.25** **±** **10**		**Total 42**	**47**		**Total 12** **+** **carcass**	**2**		
2013 (human case)	3124406222	22 August									**1**
2013	3124406342	4–11 June	28		21			4			
	3124406413	8–10 June	13		9			6			
	3124406344	11–18 June	14								
	3124406343	10–17 June	15			6					
	**Average:**		**17.5** **±** **5**		**Total 30**	**6**		**Total 10**			
2014	3124406311	9–20 July	58	2^*^	23	1	1	6		–	**3**
	3124406312	16–21 July	88		19	–	–	5			
	3124405133	19–21 July	29		–	–	–	–			
	3124405143	19–21 July	22		–	–	–	–			
	**Average:**		**49.25** **±** **23**		**Total 42**	**1**	**1**	**Total 11**			
2015	3124405233	27 July−5 August	54		20	–	–	–		3	
	3124405234	27 July−16 August	52		76					1	
	3124405243	8–11 August	30		13					1	
	3124405241	9–11 August	12		0					–	
	3124405144	5–6 August	6		11					–	
	3124406321	13–16 August	23		9					–	
	**Average:**		**29.5** **±** **15**		**Total 129**					**5**	
2016	3124406242	28 June−6 July	39		82	3		1			
	3124406241	30 June−2 July	16		0						
	3124406313	30 June−5 July	25		26						
	3124406223	2–9 July	25		0						
	3124406224	5–6 July	6		0						
	3124406232	8–9 July	29	1^*^	16						3
	3124406311	15–19 July	31		–						
	3124405043	18–22 July	9		–						
	**Average:**		**22.5** **±** **9**		**Total 124**	**3**	**–**	**1**			
2017	3124205123	15–29 June	19		7						
	3124405141	16–19 June	18		6						
	3124405052	17–21 June	13		11	2					
	3124405034	23–27 June	27		23	1					
	3124405121	27 June−6 July	34		21	1					
	3124405142	2–4 July	19		7						
	**Average:**		**21.67** **±** **5**		**Total 75**	**4**					

* *Object of isolation of Y. pestis. Bold values indicate average or total meanings*.

**Figure 2:**
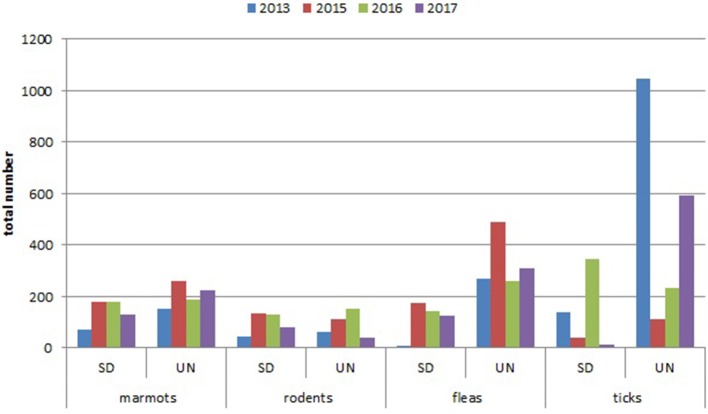
Year dynamics of plague hosts and carriers in Sari-Dzhas and Upper-Naryn plague foci (SD, Sari-Dzhas natural plague focus; UN, Upper-Naryn natural plague focus).

Comparing the total number of collected marmots in Sari-Dzhas for 2013 and 2015–2017 with that in Upper-Naryn, the last one has much higher value on the whole area observed in this period (Figure 2). At the same time, there were no differences in the total number of trapped rodents and other mammals in these periods (2013, 2015–2017) between the two plague foci (Figure 2).

### Ectoparasites

The number of ticks collected from marmots was three to five times higher than fleas collected within all sectors of both investigated plague foci (Tables 5, 7; Figure 2). In the Upper-Naryn natural plague area, the average number of fleas and ticks was significantly higher than in the Sari-Dzhas in all years, except ticks in 2016 (Figure 2).

**Table 5:** The number of “body-ectoparasites” collected from trapped marmots in open stations of the Sari-Dzhas natural plague focus in 2012–2017.

**Years of observation**	**#sector**	**Number of analyzed animal (*M. baibacina*)**	**Number of fleas**	***O. silantiewi***	***Rh. li ventricosa***	**Number of ticks**	**Number of lice**
2012	3224406334	39	19	19	–	95	**–**
	3224407512	18	43	43		60	
	3124407521	43	35	35		92	
	3124406343	26	44	44		62	
	3124406344	18	23	23		7	
	3124406433	7	22	22		1	
	3124406342	53	12	12		95	
	3124406413	25	21	19	2	36	
	**Total:**	**229**	**219**	**217**	**2**	**459**	
2013	3124406342	28	5	5	–	70	**–**
	3124406413	13	4	4	–	10	–
	3124406344	14	1	1	–	46	–
	3124406343	15				11	
	**Total**	**70**	**10**	**10**		**137**	**–**
2014	3124406311	58	15	13	2	62	2
	3124406312	88	18	16	2	81	4
	3124405133	29	1	1	–		
	3124405143	22	8	8	–	12	
	**Total:**	**197**	**42**	**38**	**4**	**155**	**6**
2015	3124405233	54	43	42	1	7	–
	3124405234	52	36	35	1	6	
	3124405243	30	7	7			
	3124405241	12	11	11			
	3124405144	6	6	4	2	5	
	3124406321	23	28	28		24	
	**Total:**	**177**	**131**	**127**	**4**	**42**	
2016	3124406242	39	90	90		91	
	3124406241	16	1	1		13	
	3124406313	25	4	2	2	105	
	3124406223	25	5	5		89	
	3124406224	6	8		8	8	
	3124406232	29	5	5		37	
	3124406311	31					
	3124405043	9					
	**Total:**	**180**	**113**	**103**	**10**	**343**	
2017	3124205123	19	12	12			
	3124405141	18	6	6			
	3124405052	13	6	6			
	3124405034	27	7	7			
	3124405121	34	8	8			
	3124405142	19	45	35	10	15	
	**Total:**	**130**	**84**	**74**	**10**	**15**	

### Isolation of *Y. pestis*

In 2012 after the 29-year period between outbreaks, one observed an acute epizootic of plague in its primary (*M. baibacina*) and secondary carriers (*C. migratorius*). In total, five strains of *Y. pestis* were isolated during the current study: two strains were from found carcasses of marmots, two were from trapped marmots, and one strain was from *C. migratorius* (Tables 3, 4). All strains were isolated from marmots trapped or found in one sector from eight studied in 2012 within the southeast part of the Sari-Dzhas focus (Figure 4).

In 2013, the human case of plague was registered on the north-west part of the Sari-Dzhas focus, sector #3124406222 (Table 4; Figure 4). In 2014, the plague epizootic in marmots with involved host-specific fleas (*O. silantiewi*) was in the neighbor sector #3124406311. Three strains of *Y. pestis* were isolated from found carcasses of marmots and fleas, collected from them, and confirmed serologically.

In 2016, the field study was conducted near the area observed in 2014–2015, with one sector overlapping between surveys (Figures 1, 4). As a result of this investigation, three strains of plague pathogen were isolated—two from *M. baibacina* (one strain from the pool inoculation of marmots captured in this area and second from a carcass of marmot found in another sector, Table 4). The third strain was isolated from pooled unidentified mites collected from a plague-positive carcass.

In 2013 and 2015, different areas of the Sari-Dzhas plague focus were surveyed with plague-negative result (Figure 1).

Three strains were isolated in the Upper-Naryn: two from marmots in 2015 and one from ectoparasites collected from marmots in 2016. All three strains were isolated in the same sector #3224407344 (Figure 4; Table 6).

**Table 6:** The number of trapped mammals in open stations of the Upper Naryn natural plague focus in 2013–2017.

**Years of observation**	**# sector**	**Time period of observation**	***Marmota baibacina***	***Microtusgregalis***	***Apodemus uralensis***	***Ochotona roley***	***Cricetulus migratorius***	***Lepus tolai***	***Cuon alpinus***	***Mustela eversmanni***	***Alticola argentatus***	***Vulpes vulpes***	**Isolated strain of *Y. pestis***
2013	3224308443	10–15 June	10	12									
	3224308434	12–17 June	28	11						1			
	3224308444	14–18 June	14										
	3224308621	19–24 June	16				4						
	3224309612	22–29 June	18				3			1			
	3224308433	28 June−2 July	16	5									
	3224309611	3–6 July	29	6			4						
	3224308431	7–12 July	23	8			2			2			
	**Total:**		**154**	**42**			**13**			**4**			
2015	3224407343	15–21 June	38	75	–	–	–	2					
	3224408513	22–28 June	62	9									
	3224408512	29 June−4 July	49	4						1			
	3224407344	5–9 July	88	19					1	1			2 from marmots
	3224407431	10–13 July	9	0									
	3224407342	11–13 July	9	0									
	3224407433	12–13 July	5	0									
	**Total:**		**260**	**107**				**2**	**1**	**2**			
2016	3224407344	7–10 June	99	112							1		1 from fleas
	3224407431	11–15 June	30	9									
	3224407342	12–17 June	10	8								1 (carcass)	
	3224407343	16–20 June	23	10									
	3224407433	20–25 June	9										
	3224408513	26–30 June	11	11									
	3224408512	30 June−5 July	8										
	**Total:**		**190**	**150**								**1**	
2017	3224408514	28 May−6 June	58	17									
	3224408522	30 May−6 June	52	15				1				1	
	3224408512	2–7 June	36	3									
	3224408523	3–10 June	51	2				1					
	3224408522	10–15 June	16	1								1	
	3224408524	16–25 June	11	–									
	**Total:**		**224**	**38**				**2**				**2**	

**Table 7:** The number of “body-ectoparasites” collected from trapped marmots in open stations of the Upper-Naryn natural plague focus in 2013–2017.

**Years of observation**	**#sector**	**Number of analyzed animal**	**Number of fleas**	***O. silantiewi***	***Rh. li ventricosa***	**Number of ticks**	**Number of lice**
2013	3224308443	10	8	4	4	45	40
	3224308434	28	8	8	–	238	43
	3224308444	14	–	–	–	76	–
	3224308621	16	4	4	–	161	44
	3224309612	18	50	39	11	159	–
	3224308433	16		5	–	95	–
	3224309611	29	39	35	4	167	–
	3224308431	23	42	19	23	99	–
	**Total:**	**154**	**151**	**114**	**42**	**1040**	**127**
2015	3224407343	38	21	21	–	1	19
	3224408513	62	99	99	–	13	–
	3224408512	49	53	51	2	60	–
	3224407344	88	62	54	8	33	13
	3224407431	9	5	5	–	–	–
	3224407342	9	2	2	–	1	–
	3224407433	5	3	3	–	4	–
	**Total:**	**260**	**245**	**235**	**10**	**112**	**32**
2016	3224407344	99	40	36	4	57	2
	3224407431	30	22	12	10	128	–
	3224407342	10	22	12	10	1	–
	3224407343	23	13	7	6	23	–
	3224407433	9	–	–	–	–	
	3224408513	11	19	9	10	13	–
	3224408512	8	10	7	3	9	–
	**Total:**	**190**	**126**	**83**	**43**	**231**	**2**
2017	3224408514	58	182	182	–	146	17
	3224408522	52	25	25	–	247	5
	3224408512	36	35	35	–	104	3
	3224408523	51	12	12	–	18	–
	3224408522	16	13	13	–	51	4
	3224408524	11	3	3	–	15	–
	**Total:**	**224**	**270**	**270**	**–**	**581**	**29**

### The Upper-Naryn Plague Focus

During 2013–2017, a total of 2,900 km^2^ were investigated in the Upper-Naryn focus, out of which we surveyed 700 km^2^ repeatedly in 2015, 2016, and 2017 (Figure 1; Table 3). In total, 828 marmots, 342 narrow-headed voles (*M. gregalis*), 13 gray dwarf hamsters (*C. migratorius*), and a small number of other mammals: six steppe polecats (*Mustela eversmanni*), four tolai hares (*L. tolai*), one red fox (*Vulpes vulpes*) were trapped and screened (Table 6). Additionally, we found the carcass of a dead fox.

From all sampled marmots, we collected and tested 1,329 fleas, 1,984 ticks, and 219 lice (Table 3). As a result, in 2015–2016, a plague epizootic was recorded in the same sector (Figure 4; Table 6). *Y. pestis* strains were isolated from *M. baibacina* and their specific fleas—*O. silantiewi*.

### Results of the Genetic Analysis

**Figure 3:**
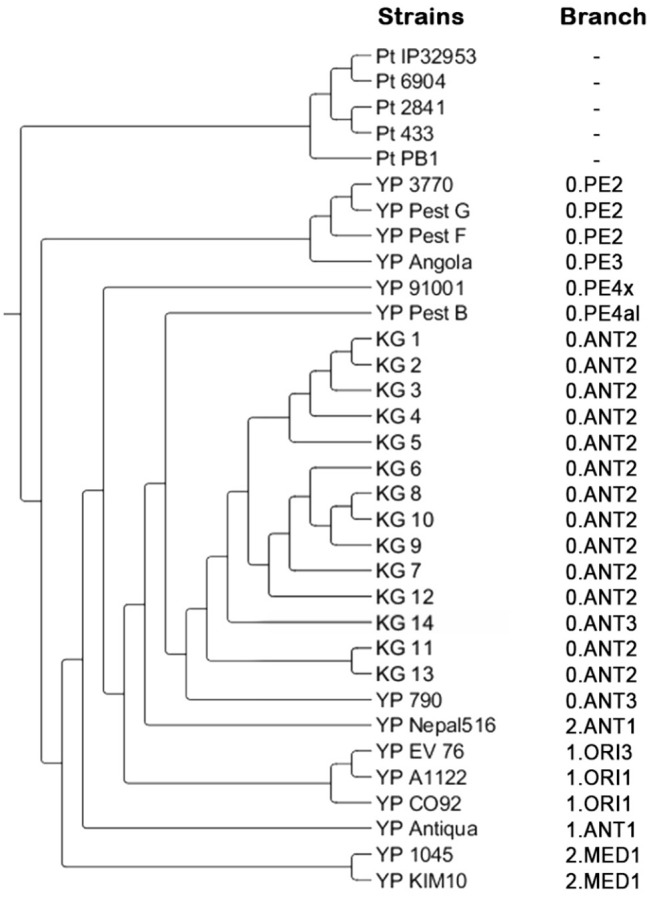
Phylogenetic tree created based on the results of MLVA and SNP analysis. Pt, *Y. pseudotuberculosis* control strains; YP, *Y. pestis* control strains; KG, *Y. pestis* strains isolated on the Kyrgyz territory of Sari-Dzhas and Upper-Naryn plague foci.

The application of the “Pest-Quest” PCR assay confirmed that all the studied strains from Sari-Dzhas plague focus belonged to the *Y. pestis* species. Based on the results of the MLVA-7 genotyping, all the studied *Y. pestis* isolates were presented by nine genotypes differing in one or several VNTR loci. Five strains (KG-1–KG-5) shared the same MLVA-7 profile (probably clones of the same strain). The software we used for phylogenetic analysis showed them not as one branch, but as a bunch of sub-branches (Table 8). All 14 strains were assigned to biovar *Antiqua* that was considered to be typical for marmot strains (Figure 3). According to the Melt-MAMA analysis, most of the strains belonged to the branch 0.ANT2, while one strain (KG-14) was apparently a member of the branch 0.ANT3 (Table 9). These two branches were among those reported by Eroshenko et al. (31) in Kyrgyzstan (along with branches 0.ANT5, 0.PE4t, and 2.MED1).

**Table 8:** Amplicon sizes of *Y. pestis* strains on 7 VNTR loci.

**Strain of *Y. pestis***	**MLVA−7**							**Genotypes**
	**ms01**	**ms04**	**ms06**	**ms07**	**ms46**	**ms62**	**ms70**	
KG_1	264	213	547	174	259	276	164	Genotype 1
KG_2	264	213	547	174	259	276	164	Genotype 1
KG_3	264	213	547	174	259	276	164	Genotype 1
KG_4	264	213	547	174	259	276	164	Genotype 1
KG_5	264	213	547	174	259	276	164	Genotype 1
KG_6	246	230	547	174	259	294	146	Genotype 2
KG_7	246	196	546	174	252	294	146	Genotype 3
KG_8	246	213	546	174	259	294	146	Genotype 4
KG_9	246	213	546	174	259	303	146	Genotype 5
KG_10	246	213	546	174	259	294	146	Genotype 4
KG_11	246	179	0	174	259	276	137	Genotype 6
KG_12	246	196	546	174	259	294	146	Genotype 7
KG_13	246	179	546	184	259	276	137	Genotype 8
KG_14	228	213	546	174	293	240	155	Genotype 9
YP_EV_76	210	230	606	184	252	222	155	Genotype 10
YP_3770	210	179	547	174	245	285	119	Genotype 11
YP_Angola	210	162	362	134	259	276	137	Genotype 12
YP_91001	210	196	303	184	252	258	128	Genotype 13
YP_Antiqua	192	179	487	164	238	231	209	Genotype 14
YP_1045	210	196	305	174	322	303	155	Genotype 15
YP_790	192	213	545	174	322	249	137	Genotype 16
YP_A1122	210	230	606	184	252	294	146	Genotype 17
YP_Pest_G	210	179	303	174	245	312	119	Genotype 18
YP_Pest_B	192	179	547	174	252	240	137	Genotype 19
YP_KIM10	192	196	305	164	378	276	146	Genotype 20
YP_Nepal516	192	196	185	174	259	222	137	Genotype 21
YP_Pest_F	210	179	487	194	245	294	119	Genotype 22
YP_CO92	228	230	606	184	252	240	146	Genotype 23
Pt_IP32953	192	145	1088	144	266	330	155	Genotype 24
Pt_6904	192	145	786	144	0	285	155	Genotype 25
Pt_PB1	192	164	606	154	301	267	200	Genotype 26
Pt_2841	174	213	0	154	252	240	128	Genotype 27
Pt_433	174	213	0	154	259	231	137	Genotype 28

**Figure 4:**
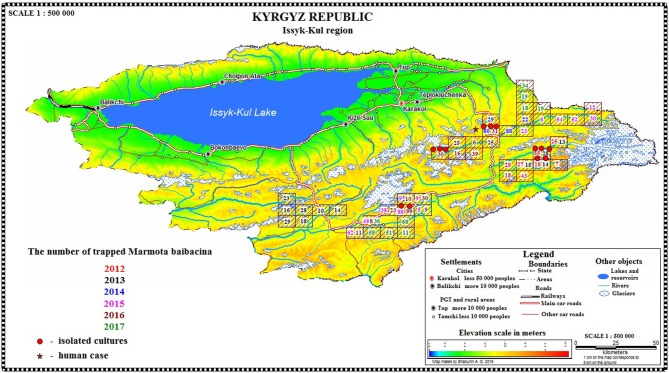
Spatial distribution of marmots per sector/year/area and isolation of *Y. pestis*.

**Figure 5:**
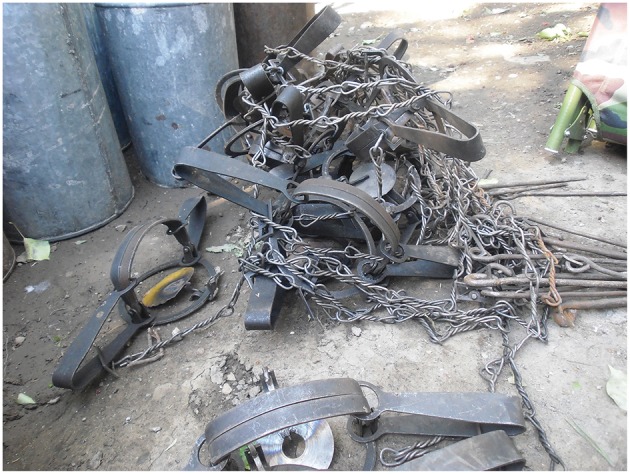
Metallic trap #3 used for trapping of marmots with chain and peg.

**Table 9:** Results of Melt-MAMA analysis.

**Strains**	**Locus s87 (T/G)**	**Locus s332 (G/T)**	**Locus s645 (G/T)**	**Biovar**	**Estimated branch**
KG-1–KG-13	G	G	G	*Antiqua*	0.ANT2
KG-14	G	T	G	*Antiqua*	0.ANT3

## Discussion

The greater part of both Sari-Dzhas and Upper-Naryn plague foci were studied simultaneously in 2013 and 2015–2017. If an epizootic of plague was observed once, the area was repeatedly examined in the succeeding years. This allows us to compare two independent plague origin areas for its epizootological status during the same time period. In the Tien Shan mountain focus of plague, the gray marmot is known as the main carrier of plague pathogen, with other rodents serving as secondary hosts (2, 8, 32, 33). The spatial distribution of marmots within plague focus is related mainly to local landscape and climatic conditions, as well as to different human activity–animal grazing, hunting, and tourism that are intensively developing during the last decade, but unequally presented in different sectors (21). Actually, the high-altitude pastures for horses and sheep (so called “syrts”) are more distant from human settlements in the Upper-Naryn area than in Sari-Dzhas. A higher distance to humans may favor the increase in density of marmots in Upper-Naryn compared to Sari-Dzhas where most of the syrts are tightly used for summer seasonal pastures and for international tourism. The disturbance of the environment may affect the marmots leading to a decrease in their population sizes. The number of other small rodents and their variability and density are approximately equal in simultaneously studied years in both plague foci. Prevalence of *M. gregalis* confirms the role of this species as a potential secondary host of plague pathogen in mixed ecosystems of high-altitude Tien Shan focus. Isolation of one *Y. pestis* strain from *C. migratorius* in the Sari-Dzhas focus confirms the role of small mice-like rodents in the epidemiology of plague. Previously, plague strains were isolated in the neighboring Aksay focus from *M. gregalis* (1968) and *C. migratorius* (1983–1984) and in the Upper-Naryn focus from *Alticola argentatus* and *C. migratorius* (3). Similar observations were reported in the high altitude of Altay and Tuva plague foci of Russia (34) and the North Aral sandy plague focus of Kazakhstan (35). Increasing density of rodents co-inhabiting with the marmots altogether with an increased number of their specific ectoparasites as observed in the Upper-Naryn focus in 2013–2017 could be a sign of potential activity of plague epizootics in this area of Kyrgyzstan. The isolation of 9 of 13 strains from marmots in 2012–2017, 1 strain from other rodents, and 3 strains from their ectoparasites is in favor of this hypothesis. Both independent plague foci (Sari-Dzhas and Upper-Naryn), located on the border of Kyrgyzstan, Kazakhstan, and China, are among the most active natural high-altitude foci of Central Asia. This territory is characterized by special climatic conditions, different relief with many heavily rugged canyons, mountain river valleys, and patches with specific flora and fauna with a high degree of biological diversity (36). Apparently, these factors give optimal conditions for the long-term circulation of *Y. pestis* biovar *Antiqua* (0.ANT2, 0.ANT3) in the populations of its natural host (31, 37). In total, 462 strains of *Y. pestis* were registered in the Sari-Dzhas area from 1944 to 1976 (4, 6, 7). Ectoparasites are actively involved in the epizootic process, in particular *O. silantiewi* and *Rh. li ventricosa* fleas specific to *M. baibacina*, as well as ixodes mites. The Sari-Dzhas area was very active in the 1940s to 1980s, and then mass disinsection of marmot populations with dichlorodiphenyltrichloroethane (DDT) insecticide and the decrease in the number of animals as a result of human activities (special extermination conducted in 1960s, hunting) led to a significant decrease in epizootic activity. Whereas, human activity (agriculture development, tourism, and hunting) changed environment during this period significantly, an obvious activation of epizootic plague activity within the area was observed after 30 years. Overall, the obtained results confirm ongoing epidemiological risk and vulnerability of the territory to plague. In such situation, it is necessary to strengthen ecological and epidemiological monitoring and control over the entire endemic area in order to preserve safety of local populations.

## Ethics Statement

All manipulations with wild and laboratory animals and handling plague strains were conducted according to the protocols approved by the Kyrgyz government regulations that are related to guidelines of Russia, Kazakhstan (1, 25). The animal work was performed according to the Regulation of the State Agency for Environmental and Forest Protection Facilities at the Government of the Kyrgyz Republic. Animal work in the field was conducted according to the following protocols: protocol 000144-KC for trapping marmots, other rodents, and analysis of their nests approved on May 6, 2014; protocol # 000168-KC approved on May 22, 2015; protocol #000173-KC approved on May 17, 2016; and protocol #000003-KC approved on March 5, 2017. Each permit to collecting a certain number of animals (400 marmots, 400 rodents of other species, and 10 nests of marmots and rodents) was issued and used during the fixed period of time (from June 1 to August 30).

## Author Contributions

GS initiated the study and wrote the first draft. GB organized and conducted the field studies and performed zoological and entomological analysis. RMu performed agreement with the state agencies for animal catching and analysis. SA provided strains for DNA analysis and genotyping. BK and AA performed molecular-genetic analysis of strains. AS performed GIS-mapping and database design. ZS performed bacteriological analysis and spatial epizootical analysis. AD performed the bacteriological analysis. ZA analyzed the environmental effect on population structure. RMa provided consultative assistance. SM provided collaborative efforts. VM edited the draft and provided reference strains. MK edited the draft and provided collaborative efforts. All authors read and approved the final manuscript.

### Conflict of Interest Statement

The authors declare that the research was conducted in the absence of any commercial or financial relationships that could be construed as a potential conflict of interest.
